# DNA2—An Important Player in DNA Damage Response or Just Another DNA Maintenance Protein?

**DOI:** 10.3390/ijms18071562

**Published:** 2017-07-18

**Authors:** Elzbieta Pawłowska, Joanna Szczepanska, Janusz Blasiak

**Affiliations:** 1 Department of Orthodontics, Medical University of Lodz, 92-216 Lodz, Poland; elzbieta.pawlowska@umed.lodz.pl; 2 Department of Pediatric Dentistry, Medical University of Lodz, 92-216 Lodz, Poland; joanna.szczepanska@umed.lodz.pl; 3 Department of Molecular Genetics, Faculty of Biology and Environmental Protection, University of Lodz, 90-236 Lodz, Poland

**Keywords:** DNA2, DNA replication, DNA repair, Okazaki fragment maturation, DNA end resection, homologous recombination repair, flap endonuclease

## Abstract

The human DNA2 (DNA replication helicase/nuclease 2) protein is expressed in both the nucleus and mitochondria, where it displays ATPase-dependent nuclease and helicase activities. DNA2 plays an important role in the removing of long flaps in DNA replication and long-patch base excision repair (LP-BER), interacting with the replication protein A (RPA) and the flap endonuclease 1 (FEN1). DNA2 can promote the restart of arrested replication fork along with Werner syndrome ATP-dependent helicase (WRN) and Bloom syndrome protein (BLM). In mitochondria, DNA2 can facilitate primer removal during strand-displacement replication. DNA2 is involved in DNA double strand (DSB) repair, in which it is complexed with BLM, RPA and MRN for DNA strand resection required for homologous recombination repair. DNA2 can be a major protein involved in the repair of complex DNA damage containing a DSB and a 5′ adduct resulting from a chemical group bound to DNA 5′ ends, created by ionizing radiation and several anticancer drugs, including etoposide, mitoxantrone and some anthracyclines. The role of DNA2 in telomere end maintenance and cell cycle regulation suggests its more general role in keeping genomic stability, which is impaired in cancer. Therefore DNA2 can be an attractive target in cancer therapy. This is supported by enhanced expression of DNA2 in many cancer cell lines with oncogene activation and premalignant cells. Therefore, DNA2 can be considered as a potential marker, useful in cancer therapy. DNA2, along with PARP1 inhibition, may be considered as a potential target for inducing synthetic lethality, a concept of killing tumor cells by targeting two essential genes.

## 1. Gene, Protein and Activity 

The DNA2 (DNA replication helicase/nuclease 2) protein was firstly identified in the yeast *Saccharomyces cerevisiae* in the early eighties and was recognized to play an important role in DNA replication both in the nucleus and mitochondria due to its helicase and nuclease activities [1,2]. DNA2 in yeast processes intermediate products of lagging strand replication in concert with the flap endonuclease 1 (FEN1). It is an essential protein as a double-knockout of its gene cannot be complemented. Its importance in yeast was underlined by a global synthetic lethal screen allowing identification of over 300 genes interacting with DNA2 and important for many fundamental processes, including DNA replication, chromatin modification and DNA repair [3]. 

**Figure 1:**
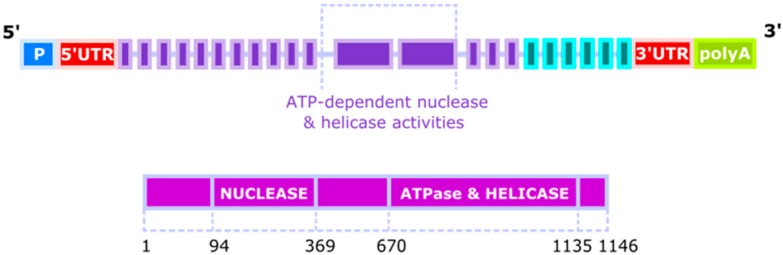
The structure of the human DNA2 (DNA replication helicase/nuclease 2) gene and protein. The *DNA2* gene has three probable alternative promoters (P), 5′ and 3′ untranslated regions (UTR) and 4 validated alternative polyadenylation sites (polyA). An approximation position of the elements of the *DNA2* gene and the DNA2 protein in aa is shown.

The gene of the human homologue of yeast *DNA2* (*hDNA2*, *DNA2L*) was mapped to the 10q21.3-22.1 chromosomal band in 1996 [4]. It has 27 distinct introns—24 spliced by canonical spliceosome and remaining 3 by its GC-AG or AT-AC counterpart. Its expression can result in at least 11 spliced variants and 3 unspliced forms in which as many as 49 exons, including 43 alternative exons and 7 cassette exons, can be found. Biochemical analyses of DNA2 revealed its ATP-dependent nuclease and helicase activities, which were associated with two separated domains (Figure 1). It is a member of the DNA2/NAM7 (nuclear accommodation of mitochondria 7) helicase family and its official name is DNA replication helicase/nuclease 2 indicating that human DNA2 might play a similar biochemical role as its yeast counterparts [5,6]. However, early studies suggested that human DNA2 was localized exclusively to mitochondria, questioning its function in the replication and repair of the nuclear genome [7]. More recent research confirmed the presence of DNA2 in mitochondria, but also revealed its activity in the nucleus, where it could play an important role in the maintenance of the nuclear genome [8]. Nuclear localization of DNA2 was observed in normal and primary cancer cells as well as in cell lineages. Human DNA2 localizes, at least in part, in the mitochondrial nucleoid, suggesting a close association of DNA2 with mitochondrial DNA (mtDNA). Only a mitochondrial localization signal was identified in the C terminus of DNA2 and not its nuclear counterpart [7]. Therefore, we do not know yet, how DNA2 is transported to the nucleus.

It has been suggested that DNA2 binds to DNA ends and separates the DNA strands that produce the substrate for its nuclease activity; this mechanism can avoid formation of too long flaps during its helicase activity [9].

## 2. DNA Replication 

Duxin et al. were the first to demonstrate that human DNA2 is present both in the nucleus and mitochondria and similar to yeast, it is an essential protein for the stability of the nuclear genome, as its absence led to chromosomal aberrations [8].

### 2.1. The Nucleus

**Figure 2:**
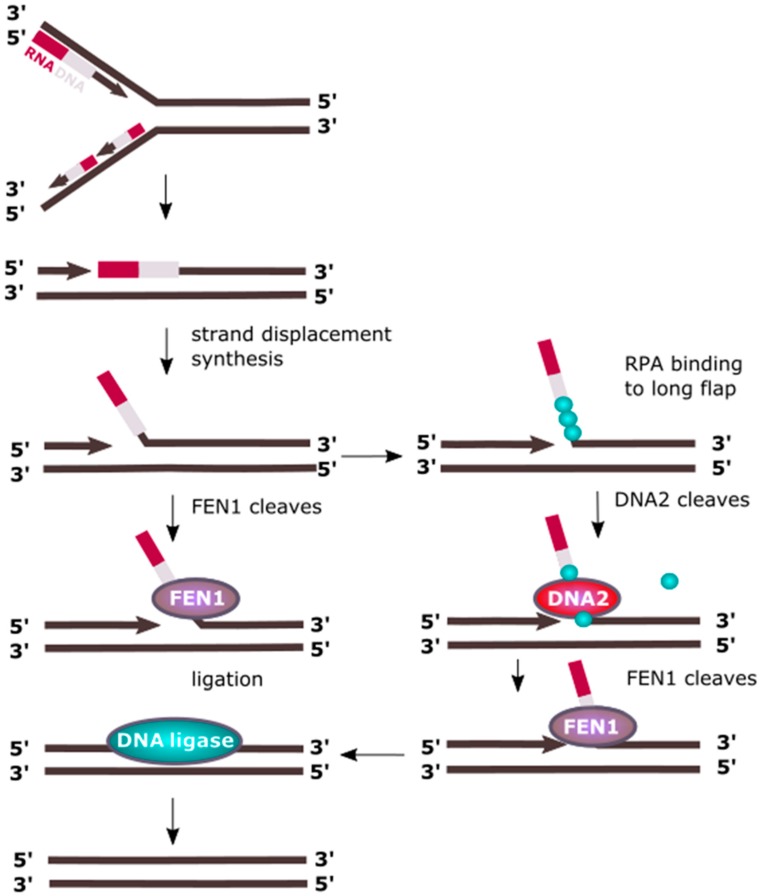
DNA2 cooperates with FEN1 (flap endonuclease 1) in processing of Okazki fragments. In eukaryotic replication, the lagging strand is synthesized in a discontinuous fashion by the production of Okazaki fragments, and synthesis each of them is initiated by an RNA/DNA primer. When DNA polymerase δ synthesizing an Okazaki fragment faces the primer of preceding fragment, it displaces it forming a flap structure, which is cleaved by FEN1. However, when the flap is too long, it is partly covered by RPA proteins blocking FEN1 and recruiting DNA2, which trims the flap, enabling FEN1 to cut it off. Okazaki fragment maturation is also possible with the involvement of an RNase, not presented here.

Processing of Okazaki fragments produced during lagging strand replication in eukaryotes includes removing the RNA component of the RNA/DNA primers and strand synthesis displacement, resulting in ssDNA (single-stranded DNA) flap structures protruding from dsDNA, which also form during LP-BER (long-patch base excision repair), as discussed in the following paragraph [10,11]. Flaps are usually relatively short stretches of DNA having about 20 nt in length and are removed by the flap endonuclease 1 (FEN1). However, RPA can bind to longer ssDNA flaps thereby inhibiting FEN1 mediated cleavage [12,13]. RPA recruits DNA2, which replaces it and cuts the flap to a length suitable for FEN1 processing (Figure 2) [14,15]. As discussed later, DNA2 can be stimulated by acetylation induced by p300, which inhibits FEN1, resulting in switching from processing of short flaps into longer ones. The latter implies synthesis of longer stretches of DNA, which apparently seems to be disadvantageous. Although the induction of longer flaps by DNA polymerase δ (Pol δ) having 3′ exonuclease activity for proofreading errors is a more energy-consuming process, it can result in a more effective removal of errors made by proofreading-less Pol α that synthesizes the DNA component of the RNA/DNA primer.

Longer flaps can also be produced by another 5′→3′ helicase, PIF1 (petite integration frequency 1) [16]. Mostly studied in yeast, PIF1 cooperates in human cells with Pol δ [17,18]. Therefore, processing of Okazaki fragments could proceed as follows: Pol δ displaces the primer of the downstream Okazaki fragment, which is further displaced by PIF1 resulting in a long flap targeted by an RPA filament. Then, DNA2 is recruited and PIF1 leaves DNA. Nucleolytic action of DNA2 results in trimming of the flap to a size suitable for FEN1, which binds DNA after disassociation of RPAs, stimulated by DNA2. FEN1 trims flap leaving a nick in the DNA, which is sealed by DNA ligase I [16,17,18]. Flap structures are recognized by the N-terminal 45-kDa domain of DNA2 [19]. 

Duxin et al. showed that the involvement of DNA2 in DNA replication can be independent of removal of Okazaki fragments [20]. DNA2 interacted with And-1 (acidic nucleoplasmic DNA-binding protein 1), a replisome protein, and this interaction depended on the cell cycle phase. Depletion of DNA2 activated the S phase checkpoint, resulting in increased number of γ-H2AX (phosphorylated variant of the H2A histone) and RPA foci as well as phosphorylation of Chk1 (checkpoint kinase 1). A reduced activation of the origin of replication was observed, which could be underlined by Chk1 activation. Depletion of FEN1 resulted in defects in Okazaki fragment maturation, but did not affect the progression of replication fork, suggesting that lagging strand processing might not strongly influence general movement of replication fork. On the other hand, DNA2-depleted cells showed neither reduction in Okazaki processing, nor disturbed progression of the fork. In conclusion, DNA2 can be involved in DNA replication through a mechanism, which is independent of FEN1 and maturation of Okazaki fragments. Equally important in that work was the observation that DNA2 depletion induced genomic instability, which could not be reversed by ectopic expression of FEN1. Therefore, that significant work suggests that DNA2 can play an important role in genomic stability by the regulation of DNA replication and checkpoint activation independently of FEN1, so the general role of DNA2 in DNA maintenance can extend assisting FEN1 in maturation of Okazaki fragments in DNA replication. 

Although DNA replication is one of the most accurate and highly regulated process, it is constantly challenged by barriers against the replication fork progression formed by DNA damage induced by endo- and exogenous factors as well as steric hindrances resulting from changes in secondary DNA structure and the presence of protein involved in DNA metabolism [21]. These complications can lead to arrest of the replication fork or its damage resulting in replication arrest. In some cases, the action of the fork can be reversed, which can be seen as a general pathway of replication reactivation, but its mechanism is not fully known [22]. This process is initiated by replication fork regression, where the newly synthesized DNA on the leading and lagging strands anneal to form a Holliday junction, termed as ‘chicken-foot’ structure that is stabilized by annealing helicases such as SWI/SNF-related, matrix-associated, actin-dependent regulator of chromatin, subfamily A-like 1 (SMARCAL1) [23,24,25]. Thereafter, the regressed replication fork could reinitiate after the DNA repair machinery has cleared the DNA lesion [26]. Alternatively, the replication fork can collapse with dissociation of the replication machinery, followed by processing of the reversed fork and repair of the one-ended DSBs (DNA double-strand breaks) by homologous recombination repair (HRR) [27,28,29,30]. Therefore, reversal of the replication fork can save the replication, but on the other hand restart of arrested replication requires processing of reversed fork [31]. To resolve that four-junction DNA structure the activity of a DNA helicase is required and the RECQ1 (ATP-dependent DNA helicase Q1) helicase was reported to promote the restart of replication fork arrested by the inhibition of DNA topoisomerase I (TOP1) [32].

**Figure 3:**
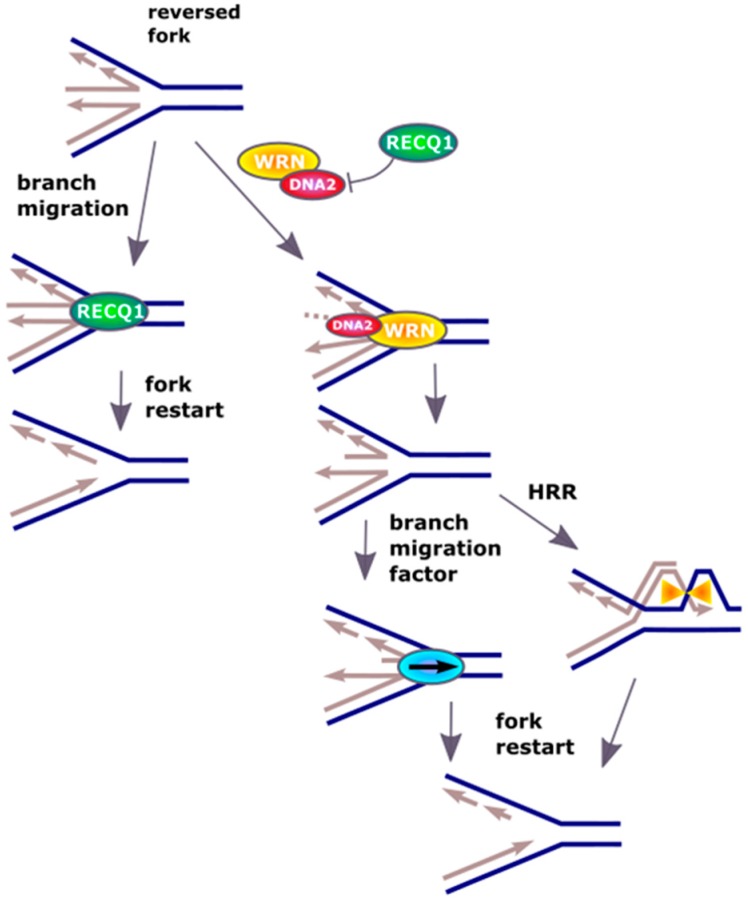
DNA2 restarts reversed replication fork. Excessive unwinding of the template in DNA replication, resulting from escape of DNA helicase from DNA polymerase or DNA damage in the template, leads to regression of replication fork by pairing of the newly synthesized strands. Resulted “chicken foot” structure is primarily resolved by the RECQ1 helicase, but if its activity is inhibited or in RECQ1-deficient cells, DNA2 can cleave the single-strand 5′ end of the regressed fork assisted by ATP-ase activity of WRN (Werner syndrome ATP-dependent helicase) or BLM (Bloom syndrome protein) (not presented). The restart is preceded by the action of branch migration factors, not displayed in that scheme. Recombination mechanisms based on homology (HRR) are an alternative for RECQ1 and DNA2 action.

The effect of RECQ1 helicase is not the only mechanism restarting reversed replication fork and it was shown that DNA2 could contribute to this process even in RECQ1-deficient cells [33]. This protein used its nuclease activity to degrade reversed fork and restarted replication in cooperation with ATPase activity of the Werner syndrome ATP-dependent helicase (WRN) (Figure 3). WRN is a homologue of RECQ1 and can assist DNA2 in end resection [34,35]. A similar function can be displayed by Bloom syndrome protein (BLM). It is suggested that WRN and BLM can act epistatically with DNA2 in end resection during DSBs repair [35]. The replication-restarting effect of DNA2 was observed for different treatments resulting in replication stalling, including an inhibitor of ribonucleotide reductase, an inhibitor of TOP1 and a cross-linking agent. Experiments with RAD51 (RAD51 recombinase) depletion showed that replication restarting activity of DNA2 was directly associated with restart of the fork. The exact mechanism of DNA2 involvement in that effect is not completely known, but some speculation can be made. DNA2, assisted by WRN, produces ssDNA fragments at the termini of reversed fork. These single-stranded overhangs can initiate HRR, which results in the invasion of dsDNA in front of the fork producing a branch, which then migrates partly restoring the replicative functionality of the fork. Branch migration can be recognized by specific proteins, including a RecQ helicase, assisting in the restart of the fork.

Our knowledge on all interactions of DNA2 with components of replication machinery in the nucleus is far from complete, but there is strong in vitro evidence for the interaction of this protein with PCNA (proliferating cell nuclear antigen), which is a master in DNA replication and plays a crucial role in several DNA repair pathways [36]. 

### 2.2. Mitochondria

TWINKLE is a mitochondrial DNA helicase involved in the replication of mtDNA [37,38]. Its mutated form induces the arrest of mtDNA replication, replication fork disintegration and eventually mtDNA degradation [39,40,41,42]. Therefore, cells with a defective TWINKLE can be a model for studying the involvement of other proteins in mtDNA replication. It was observed that DNA2 colocalized with TWINKLE in cells with its normal expression. However, in cells with mutations in TWINKLE causing stalling of replication fork, DNA2 changed localization and colocalized with the mitochondrial nucleoid proteins, suggesting its role in mtDNA replication [8]. This role was confirmed by a decreased production of replicative intermediates in the form of bubble and y-type arcs and RITOLS (ribonucleotide incorporation throughout the lagging strand) structures in cells with depleted DNA2 [8].

Zheng et al. showed that DNA2 directly interacted with Pol γ, the mitochondrial replicase [7]. They showed that the association of DNA2 with Pol γ dramatically increased the ability of primer extension in an in vitro model of mitochondrial replication as compared with the action of the polymerase only. That model assumed that mtDNA replication occurred via a strand-displacement mode with the formation of three-stranded D-loop [43,44,45]. Pol γ singly synthesized fragments shorter than 50 nt, but its association with DNA2 resulted in extension of the product up to 400 nt and an approximate 60-fold increase in the overall activity of the enzyme. This increase was attributed to DNA2 helicase activity causing displacement of duplex mtDNA during its replication. Therefore, DNA2 can play a role of a replicative helicase as it can be at least as important as TWINKLE for mtDNA replication [46].

**Figure 4:**
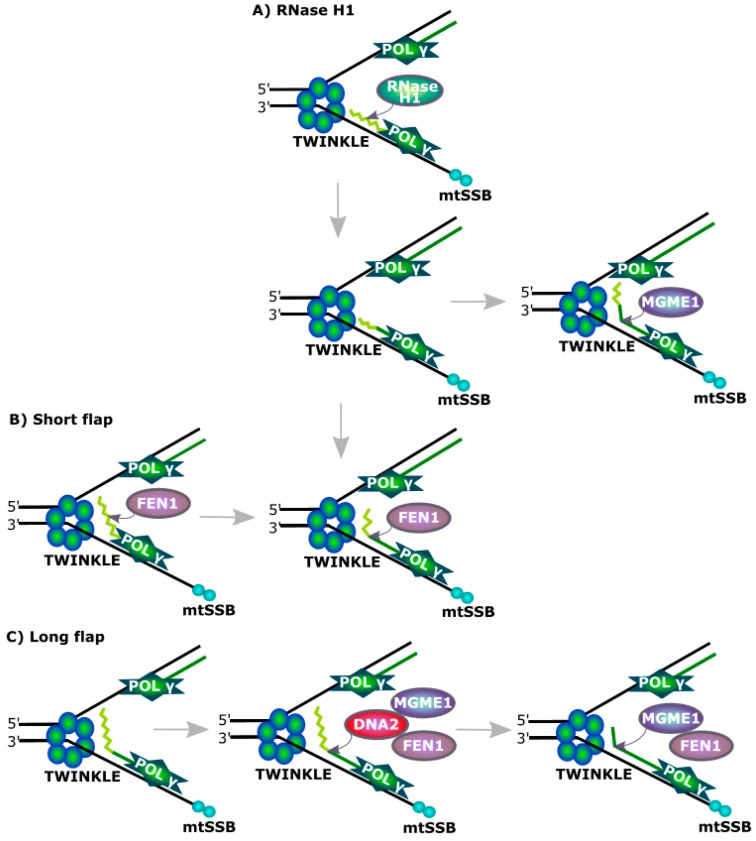
Primer removal during mitochondrial DNA replication. Progress of replication fork is mediated by a DNA topoisomerase (not presented here) separating intertwined mtDNA and the TWINKLE helicase. RNase H1 can degrade most of an RNA primer, but it is not able to remove its last ribonucleotide. When this fragment is reached by Polγ, it is displaced as a short flap, which can be degraded by FEN1 or MGME1 (mitochondrial genome maintenance exonuclease 1). FEN1 can cleave away entire RNA primer, when it forms a flap and is not too long, but during replication of a heavy strand of mtDNA, primers are much longer than those of its light counterpart and FEN1 cannot do so. In that case, a coordinated action of FEN1 and DNA2 or MGME1 preceded by FEN1 can occur. mtSSB is a mitochondrial single stranded DNA-binding protein.

DNA2 was shown to be involved in the removal of RNA primers during the replication of mtDNA (Figure 4). However, FEN1 is an endonuclease specializing in processing flap structures and it can remove RNA primers from replicating mtDNA alone, but its association with DNA2 results in increased efficiency of this process, which can be assisted by RNaseH1, recognizing a DNA-RNA hybrid and cleaving its RNA component [47,48]. However this enzyme is not able to remove the last ribonucleotide in the degraded flap structure as it is bound by a phosphodiester bond with a deoxyribonucleotide. This critical for RNaseH1 nucleotide can be removed by either FEN1 or DNA2, with 5′ exonuclease activity. The MGME1 (mitochondrial genome maintenance exonuclease 1, Ddk1) protein is another factor of primer removal in mtDNA replication [49,50].

DNA helicase activity is critical for the replication of mtDNA and it is primarily delivered by TWINKLE. However, DNA2 is not the only other DNA helicase as PIF1, suppressor of var1 3-like (SUV3) and RecQ-like helicase 4 (RECQ4) can be found in an active state in mitochondria [32].

## 3. DNA Damage Response

### 3.1. Post-Translational Modifications

Post-translational modifications of DNA2 are important for its functions. Acetylation of this protein by p300 stimulates its 5′→3′ helicase, 5′→3′ endonuclease and DNA-dependent ATPase activities [51]. It is important to note that p300-mediated acetylation of FEN1 inhibits its DNA binding and nuclease activity indicating a p300 regulated coordination between FEN1 and DNA2 during DNA replication and base excision repair (BER), as discussed later [52,53]. 

Another important post-translational modification of DNA2, which can be crucial for its DNA resection functions is phosphorylation. DNA end resection, a prerequisite to HRR and other pathways of DNA repair, depends on the cell cycle regulation [54]. Cyclin dependent kinases (Cdks) are the master regulators of the cell cycle and they are also involved in DDR regulation [55]. In light of results obtained so far it seems that this is Cdk1, which is the closest connection between end resection, HRR and cell cycle [54,56]. Moreover, Cdk1 is needed for the initial stage of DSB repair by HRR [57,58]. 

Chen et al. reported that the resection of DNA ends at DSBs by yeast DNA2 was stimulated by a specific phosphorylation of DNA2 [59]. In general, in yeast, the initial stages of DSB-induced HRR, primarily end resection, are controlled by Cdk1. These authors showed that the involvement of DNA2 in DNA long-range resection depend on Cdk1. That protein along with Mec1, a budding yeast homolog of ATR (ATR serine/threonine kinase), phosphorylated DNA2 to activate its resection action. Further, they showed that Exo1 could assume the role of DNA2 when it is mutated, providing evidence of crosstalk between DNA2 and Exo1. Finally, they showed that only a specific pattern of Cdk1-induced phosphorylation stimulated DNA2 recruitment to DSB sites and its resection action. Namely, phosphorylation of Ser17 and Ser237 was essential for nuclear entry and DSB resection by DNA2, whereas mutants with substitutions in these residues or deletion, were weakly recruited to DSB sites and promoted resection only in the presence of Exo1. 

In summary, post-translational modifications of DNA2, especially its specific phosphorylation induced by Cdk1 and acetylation, can be critical for essential functions of DNA2–DNA replication and DNA repair. However, most of these studies were performed in yeast, so their validation in humans is needed. Moreover, as acetylation and phosphorylation are not the only post-translation modifications, other kinds of changes imposed on DNA2 after translation should be checked if they influence DNA2 functions. 

### 3.2. Cell Cycle Regulation and Telomere Maintenance

In yeast, chromosomal localization of DNA2 depends on the cell cycle [60]. The protein was found in telomeres during G1, late S and G2 phases, but during DNA synthesis, it localized in the regions of chromosome replication. In late S and G2, DNA2 was involved in telomere replication and telomerase-dependent elongation. Additionally, DNA2 relocated from telomeres to DSBs after treatment with bleomycin, an anticancer drug inducing a broad spectrum of DNA damage including DSBs. This suggests the involvement of DNA2 in telomere maintenance, which is critical for genome stability [61]. 

Telomeres contain ssDNA, which is rich in guanines—in humans, telomeric DNA has many copies of the 5′-TTAGGG-3′ motif [62,63]. Guanines can self-assembly forming guanine quartets (G4s) or tape-like G-ribbons [64]. In consequence, single-stranded G-rich telomeric DNA can fold onto itself forming four-stranded DNA, which can hinder DNA replication as such structure is resistant to most helicases and nucleases processing replication intermediates. It was shown that yeast and human DNA2 bind G4 with much higher affinity than telomeric ssDNA [61,65]. Therefore, the role of DNA2 in telomere maintenance can include resolution of telomeric G4s. On the other hand, G4 inhibited the nuclease activity of DNA2, which was partly restored by RPA, but RPA inhibited the DNA2 3′→5′ nuclease activity [65].

Depletion of DNA2 in osteosarcoma U2OS and HeLa cells resulted in an accumulation of these cells in the G2/M checkpoint, likely following from chromosomal aberrations, which in turn was a manifestation of general genomic instability [8]. 

### 3.3. DNA Repair

As mentioned, DNA2 in yeast in S phase localized in the regions of replicating chromosomes and in resting phases—at telomeres, but when the cells were exposed to the DNA-damaging drug bleomycin, the protein translocated to the sites of DNA damage [54]. This implies the involvement of DNA2 in both DNA replication and repair, but also suggests that DNA repair by DNA2 might be associated with replication. In other words, it would be possible that the participation of DNA2 in DNA repair does not follow from the fact that it is a DDR protein, but rather a protein assisting replication and preventing its impairments. However, in yeast, DNA2 mutants were oversensitive to ionizing radiation and alkylating agents, suggesting an involvement of DNA2 in DNA double-strand break repair (DSBR) and BER or mismatch repair (MMR) [66,67]. 

DNA double-strand breaks and DNA interstrand cross-links (ICLs) are the most serious DNA damage, which can result in chromosome breakage and translocation [68]. Two basic mechanisms of DSBs repair in humans are non-homologous end joining (NHEJ) and HRR [69]. Although NHEJ is frequently considered as a preferable pathway of DSBR in higher eukaryotes, it is error-prone and can result in loss of genetic information [70]. HRR requires 3′ single-stranded fragments (3′ overhangs), which are produced in DNA end resection. This process is usually catalyzed by a combined action of DNA helicase and 5′→3′ nuclease. The resulting 3′ ss overhangs are targeted by HRR proteins. In yeast, resection requires a join action of Sgs1 (slow growth suppressor 1) and DNA2 as a nuclease [66]. Another pathway involves exonuclease I (ExoI), a 5′→3′ exonuclease, which is specific to dsDNA [71]. 

In general, the process of DNA end resection can be divided into two steps: resection initiation and resection extension [72]. The former is stimulated by SRCAP (Snf2-related CREB-binding protein activator protein), CtIP (C-terminal binding interacting protein) and MRN complex, whereas the latter is promoted by SMARCAD1 (switch/sucrose non-fermentable (SWI/SNF)-related matrix associated actin-dependent regulator of chromatin subfamily A containing DEAD/H box1) cooperating with EXO1 and the BLM/DNA2 complex [73].

In humans, there are five homologs of RecQ—RECQ1, RECQ4, RECQ5, WRN and BLM and the latter was reported to participate in DSB resection [74,75,76,77]. RPA, a single-strand binding protein, is loaded onto 3′ overhangs and recruits RAD51, the key protein of HR, with the involvement of BRCA2 [78,79]. It should be stressed that MRN is involved in the early detection of DSB and is an executor of DNA ends resection, as discussed later [80,81,82]. EXO1 and BLM can act in combination, but such action is non-essential [83]. However, Nijmonkar et al. showed that BLM and DNA2 formed a functional complex resecting DNA in the 5′→3′ direction in the presence of RPA [84]. This complex acts in a highly coordinated fashion—BLM acts as a DNA helicase and DNA2 resects unwound DNA. The nucleolytic action of DNA2 in the 5′→3′ direction is stimulated by RPA, but as mentioned, RPA attenuates DNA2 nucleolytic activity in the reverse direction. Besides RPA, MRN stimulates resection by BLM–DNA2 complex, mainly by a more effective recruiting of BLM to resected DNA. Therefore, two kinds of complexes responsible for resecting DNA ends resulting from DSB can be considered: BLM-DNA2-RPA-MRN and BLM-EXO1-RPA-MRN. The choice between them is not completely known, but structure of the broken DNA ends can play a role. 

A DNA single-stranded 3′ overhang produced by resection activity of either BLM-DNA2-RPA-MRN or BLM-EXO1-RPA-MRN serves as a substrate for an invasion of a DNA molecule in searching for homology partner for HRR. In humans, the RAD51 protein promotes invasion, homology search and strand exchange to form a new DNA duplex. As BLM is reported to directly interact with RAD51, it is a candidate to direct RAD51 to ssDNA produced by resection by either complex [85]. Damage in replicating DNA can be converted into a DSB, which stalls the replication fork [36]. Resulting DSB ends can be resected with nuclease activity of DNA2 and then targeted by RAD51 and other recombinational repair proteins, whose action results in restart of the stalled fork.

Recently Miller et al. reported that budding yeast cells lacking Exo1 required DNA2 helicase activity to perform a DNA end resection reaction [86]. DNA2 cleaved a 5′ flap located in the vicinity of an ssDNA/dsDNA junction when translocated on ssDNA with its ATP-stimulated translocase activity. This effect was associated with an inhibition of 3′ flap endonucleolytic activity of DNA2.

Tkac et al. reported another important player on the scene of DNA end resection—DNA helicase B (HELB) [87]. HELB negatively regulates DNA end resection by inhibiting DNA2-BLM or EXOI in human cells. When DNA2 complexed with BLM resect DNA, it can be inhibited by 5′→3′ ssDNA translocase activity of HELB. This mechanism requires the involvement of RPA. Importantly, HELB did not inhibit the flap nuclease activity of DNA2, which was not complexed with BLM. Therefore, the involvement of DNA2 in resection reaction can determine it as a substrate for the action of other proteins, so its own activity is regulated in dependence on its association in functional complexes with other protein(s), in that case—with BLM. However, during the 5′ DNA end resection, BLM acts in complex with DNA topoisomerase IIIα (TOPIIIα) and RMI1-RMI2 (RecQ mediated genome instability 1–2) proteins [88]. DNA2 stimulates the helicase activity of BLM and TOPIIIα can enforce 5′→3′ directionality of DNA2 resection by DNA2 [85]. Moreover, TOPIIIα attenuated the 3′ endonuclease activity of DNA2 and importantly TOPIIIα was suggested to be specific for the BLM-DNA2 pathway of DNA end resection, whereas MRN stimulates both BLM and EXOI [84,89].

Disturbed action of DNA topoisomerase 2 (TOP2) as well as some other DNA processing enzymes, can result in a complex DNA damage in the form of DSBs associated with 5′ adducts [90]. In the case of TOP2, such adducts are formed by two subunits of the enzyme covalently linked to both 5′ ends in double-nicked DNA. These adducts are trapped by cancer drugs and converted into DSBs with 5′–TOP2 adducts. Such complex DNA lesions are usually processed by more than one DNA repair pathway and detailed mechanism of it is not exactly known, but it is critical for cell survival. Tammaro et al. showed that DNA2 was critical in end resection of DNA treated by etoposide, a DSBs inducing anticancer drug [91]. Therefore, DNA2 can be a major molecule in the repair of this kind of complex DNA damage.

In eukaryotic cells, all DNA processing occurs within their nucleosomal organization and so end resection requires access to DNA within chromatin. It was shown that the Fun30 (function unknown now 30) protein, which is a member of Swi2/Snf2 (switch/sucrose non-fermentable) family of proteins involved in chromatin remodeling, can facilitate the action of DNA2 in end resection by overcoming inhibiting effects induced by Rad9, which otherwise create a barrier for the resection [92]. The potential human homologue of Fun30 is already presented SMARCAD1, which was shown to be involved in end resection and colocalize with DSB repair markers, including γH2AX [73,93].

DNA double-strand breaks and interstrand-crosslinks are the most serious types of DNA damage. The latter are of special significance as there is not a single DNA repair pathway dedicated to process them, but they are corrected with a combination of different DNA repair mechanisms [90]. Encountering of an ICL by replication fork can lead to its disintegration, reversion and DSBs. The Fanconi anemia (FA)/BRCA (breast cancer) pathway was identified to play a fundamental role in ICL repair and the rescue of ICL-related stalled replication fork [94,95,96]. DSBs can appear as a consequence of ICLs in replicating DNA or can be produced as an intermediate of ICL repair. As mentioned, DSBs can be repaired mainly by two repair pathways: NHEJ and HRR, but the choice between them is not completely known. However, if end resection occurs after DSBs induction, it will be a good prerequisite for HRR. The process of end resection increases the affinity of a key FA/BRCA regulatory complex FANCD2-FANCI to DSBs site. As DNA2 is involved in end resection, it can interact with this complex or in general—with the FA/BRCA or FA pathway. Therefore, DNA2 can be a key player in ICL repair although mechanistically this role is probably limited to processing of intermediates of ICL repair—DSBs, resulting from the double incision of one DNA strand, externalization of the crosslink (“unhooking”) and excision it from the other DNA strand, as shown by Karanja et al. [97,98]. These authors also appreciated the role of DNA2 in the S checkpoint activation. They hypothesized that DNA2 might act downstream of FANC2 and upstream of BRCA2 in the FA/BRCA network, which suggests that DNA2 can be involved in homology-directed DNA repair. HRR is the most common mechanism of such repair, but a mechanism, which does not involve strand invasion, involves strand resection and is biochemically distinct from HRR, single-strand annealing (SSA), can also play a role. Karanja et al. showed that DNA2 depletion reduced SSA in human cells. They also showed that NHEJ, a dominant DSB repair pathway in S/G2, is induced in DNA2-depleted cells as demonstrated by an increased level of catalytic subunit of DNA-dependent protein kinase (DNA-PK_CS_), a key protein for canonical NHEJ. The importance of DNA2 for DSB repair is underlined by the accumulation of DSBs in DNA2-depleted cells, evidenced by elevated level of γH2AX/RAD51 foci and Chk1 accumulation [8].

In their subsequent research, Karanja et al. showed that DNA2 depletion in normal cells made them as sensitive to crosslinking agents as cells with double knockout in the *FANCD2* gene, required for crosslinks repair in the FA pathway [98]. However, depletion of DNA2 in *FANCD2*-deficient cells rescued their sensitivity to agents inducing DNA–DNA and DNA–protein crosslinks, which are repaired in the FA pathway. This suggests antagonistic roles of DNA2 and FANCD2 and a devastating potential of DNA2 in DNA repair, which is underlined by its increased resection activity. Therefore, a dual role of DNA2 in the maintenance of genomic stability may be also considered. However, this is not very surprising, as in general the activity of all DNA repair proteins should be adjusted to cooperate in DNA repair execution. 

Depletion of DNA2 resulted in a decreased efficacy of the repair of oxidatively damaged mtDNA induced by a high concentration of hydrogen peroxide as measured by quantitative PCR in HeLa cells [8]. Base excision repair in mitochondria is often considered as the main DNA repair system operating in this organelle [99,100]. Similar to the nucleus, BER in mitochondria can be divided into two subpathways: short-patch BER (SP-BER) and long-patch BER (LP-BER), and the latter includes the production of a flap intermediate, which must be removed before sealing remaining nicks by a DNA ligase. Both FEN1 and DNA2 can participate in removing this intermediate, and they mutually stimulate their activities as they displayed a synergistic effect. One of possible mechanism underlying such an effect can be stimulation of the nucleolytic action of FEN1 by DNA2 helicase activity. Similar mechanisms occur also in single-strand break repair (SSBR), a DNA repair pathway which is not initiated by a DNA glycosylase whose exact mechanism in mitochondria is not known [101,102]. Therefore, DNA2 can play an important role in LP-BER, mechanism for which partly overlaps with DNA single-strand break repair (SSBR). However, it seems not to be indispensable in this process, as another 5′exo/endonuclease, EXOG, was identified [103]. This protein is localized exclusively in mitochondria, displays 5′→3′ exonuclease activity and its action can be critical for mtDNA maintenance, as its depletion resulted in accumulation of SSBs, increased oxidative stress and apoptosis induction.

## 4. Cancer

Many cancers are associated with oncogene activation, which can result in replication stress, and DNA2 plays an important role in cellular defense against this stress. As mentioned, this role is mainly expressed by DNA ends resection, which is a prerequisite for HRR-directed repair of DSBs resulting from replication stress. Therefore, inhibition or depletion of DNA2 can result in lower survival of cancer cells facing oncogene-activated replication stress. Homologous recombination repair-promoting effects of DNA2 preventing accumulation of replication-related DSBs can be related to carcinogenesis, as oncogenes can promote replication stress resulting in DSBs. Peng et al. showed that DNA2 formed a complex with replication factors decreasing the number of DSBs associated with replication in cell line with forced expression of the H-RAS (Harvey rat sarcoma viral oncogene homolog) oncogene [36]. This raises the question about the clinical significance of DNA2 in cancer. Cancer microarray data identified enhanced expression of DNA2 at mRNA and protein levels in cancer cells [104,105]. Enhanced DNA2 expression was also observed strongly in cell lines with atypical hyperplasia, a premalignant state typical for many cancers [36]. It was observed that enhanced expression of DNA2 occurred in an early stage of cancer transformation in a cell model with K-RAS (Kirsten rat sarcoma viral oncogene homolog) activation [106]. Moreover, increased DNA2 expression was positively correlated with ovarian cancer grading [36]. This enhanced DNA2 expression was associated with increased genomic instability typical for almost all cancer cells. 

It was shown that DNA2 was significantly overexpressed in pancreatic cancer cell lines, and its inhibition resulted in a lower survival of cancer cells and a decreased growth of their xenografts [107]. A panel of DNA2 inhibitors was developed with effects compared to those obtained by genetic depletion of DNA2. These inhibitors were effective in cells with overexpressed H-RAS and K-RAS oncogenes. Therefore, a new target in cancer therapy can be considered—DNA2, whose inhibition can result in eradication of cancer cells following from their decreased ability to repair DSBs produced by oncogene-activated replication stress. 

Genomic instability is associated with defects in DNA repair resulting from mutations in DNA repair genes. These defects can lead to inactivation of a particular DNA repair pathway, naturally functioning in normal cells. However, the functions of affected DNA repair pathway can be taken over by another (alternative) pathway if they are characterized by the same or similar specificity of substrates. If there is no other way to repair a certain class of DNA damage than these two pathways, a deliberate inhibition of the alternative pathway can result in a complete inability of a cancer cell to repair that class of DNA damage. This is an example of the concept of synthetic lethality—two genes are mutually lethal if mutations in both of them result in a cell death, but if only one of them is mutated—the cell survives [108,109]. Breast cancer cells with mutations in the *BRCA1* and *BRCA2* genes can display defects in DSBs repair in HRR pathway and inhibition of PARP1 (poly(ADP-ribose) polymerase 1), which is a crucial protein in SSBR, can result in death of these cells [110]. Unrepaired SSBs can lead to apoptosis either by their conversion to DSBs or by replication fork collapse at these SSBs. As DNA2 can play an important role in HRR of DSBs, its defects can justify the use of PARP1 inhibitors [36]. As mentioned, HELB negatively regulates the process of DNA resection, so it is not surprising that loss of its function results in resistance to PARP-1 inhibitors in cancer cells deficient in BRCA1 [83]. DNA2 can, at least functionally, cooperate with PARP-1 in DNA repair and restoring disturbed DNA replication [111,112,113].

Human cells require a DNA helicase to proliferate, so the lack of replicative helicase as DNA2 can lead to diseases associated with aberrant cell proliferation. Moreover, genetic dysfunction of DNA2 can be transmitted to next generations, resulting in a hereditary disease. This is supported by well-known associations of mutations in DNA helicase gene and severe cancer-prone familial diseases, including Xeroderma pigmentosum and Bloom syndrome [114,115].

Mutations in the *DNA2* gene were associated with a form of mitochondrial myopathy linked with genomic instability in muscle mtDNA [116]. Three heterozygous missense mutations in the *DNA2* gene c.851G > A, c.937A > G and c.2167G > A were identified. The effect of these mutations in muscle cells could be associated with an impaired function of DNA2 in the repair of oxidatively damaged DNA in long-patch BER, which could result in genomic instability in these cells. As mentioned, genetic instability is a feature of cancer cells and cells with this feature can be considered as cancer-prone although they can express a disease not related to cancer.

Deregulation of helicase action in DNA replication can also result in increased genomic instability. The *DNA2* gene was reported to be mutated in estrogen-dependent breast and ovarian cancers [117]. These mutations were found mostly in the domains encoding helicase and nuclease activity of DNA2. However, the overall expression of DNA2 in breast cancer MCF-7 line was increased when treated with estrogen. This overexpression, which might be associated with amplification of the *DNA2* gene, is associated with a poor survival of estrogen-dependent cancer patients. Therefore, DNA2 can be considered as a molecular target in such cancers, both directly and in synthetic lethality. 

As DNA repair and DNA replication are essential for cell survival and growth, their inhibition in cancer cells results in cytotoxic or cytostatic effect. Therefore, DNA2 as essential for both processes, can be considered as a target in anticancer therapy. It is of a special significance as cancer cells frequently upregulate proteins for their growth. Liu et al. showed that homozygous deletion of the *DNA2* gene increased the sensitivity of cancer cells to radiation and camptothecin (CPT), an inhibitor of DNA topoisomerase I [118]. Using a high throughput screen, they identified a selective and effective inhibitor of DNA2—4-hydroxy-8-nitroquinoline-3-carboxylic acid (C5), which restored CPT sensitivity in DNA2 overexpressing cells. 

The *TP53* (tumor protein p53) gene is frequently mutated in cancer and these mutations can be associated with gain of function(s) [119,120]. DNA2 depletion in cancer cells with gain-of-function mutant form of p53, induced their hypersensitivity to anticancer drugs [121]. Therefore, many effects of DNA2 predispose it as a useful marker of cancer transformation and target in anticancer therapy.

## 5. Conclusions and Perspectives

It is not easy to answer the question asked in the title of this review. There is no doubt that DNA2 is important for completing DNA replication and plays a significant role in DNA repair in general. Therefore, it is not “just another DNA maintenance protein”. It is essential in replicating cells. In DDR it plays an important role in DNA repair, mainly in homologous recombination repair, dealing with double-strand breaks. Its leading function in HRR is resecting DNA ends preparing them for invasion for homologous duplex. However, the role of DNA2 in DSBs repair is not limited to end resection. HHR and NHEJ are the main pathways of DSBs processing in human cells, but the regulation of the choice between them is still poorly known, despite it is a fundamental problem as these two DNA repair systems can produce substantially different DNA molecules. It can be speculated that the structure of DNA ends can play a role in the choice between HRR, NHEJ and other DSBR pathways. Therefore, as DNA2 can be essential for end processing, it can be indirectly involved in the choice between NHEJ and HRR. Even if one stresses that this choice is mainly determined by the cell cycle regulation mechanisms, DNA2 can be considered as a potential candidate in this regulation as it expression was dependent on the cell cycle phase. This review is mainly focused on human DNA2, but in this moment it can be mentioned that a DNA2 homologue was observed to regulate cell cycle progression in plant meristems [122]. We think that the determination of a role of DNA2 in the regulation of the choice between HRR and NHEJ belongs to the most important directions of future research on this protein.

However, a more specific question is about the role of DNA2 in the choice between complexes responsible for resecting DNA ends in DSBs: BLM-DNA2-RPA-MRN and BLM-EXO1-RPA-MRN. This leads to the conception of competition between DNA2 and EXOI and structures and accessibility of DNA ends can be considered as primary determinants of this competition. 

In mitochondria, DNA2 can act in concert with FEN1 to facilitate primer removal during strand-displacement replication and increase the efficacy of Pol γ, the mitochondrial replicase.

The main role of DNA2 in DNA repair seems to be the resection of DNA ends in DSBR. The same mechanism underlines the role of DNA2 in BER and SSBR, in which DNA2 processes flap structures in repaired DNA, similarly to processing of intermediates of DNA replication. Therefore, the mechanism of the involvement of DNA2 in DNA replication and repair is somehow limited, but this protein has just two essential enzymatic activities, nuclease and helicase, supported by ATPase. It should be stressed that DNA2 can recognize DNA damage *per se*, like in the case of DSB, so its activity is not limited to the response to a specific structure in intermediates of DNA repair, as does FEN1. 

The involvement of DNA2 in several DNA repair pathways and DNA replication as well as its connection with cell cycle regulation and telomere maintenance suggests its important role of this protein in maintaining genomic stability. Because genomic instability is a feature of most, if not all, cancer cells, DNA2 can play a role in cancer transformation, which makes it a candidate for a target in anticancer therapy. Initial experiments justify undertaking further research in this subject. 

Chemicals stabilizing TOP2–DNA cleavage complex are frequently used as anticancer drugs and they representatives are various anthracyclines and etoposide and other TOP2 poisons. Their action results in DSBs and DNA-protein formation, a complex DNA damage, which is repaired by a complex, not fully-known mechanism, but DNA2 was suggested to be critical for it. Again, there is a problem to be solved, whether the role of DNA2 in that repair was limited to DNA end resection or was beyond it. The answer can be important at least for two reasons, Firstly, our knowledge about the mechanism of repairing of complex DNA lesions is seriously limited and further exploring of the role of DNA2 could bring important information on it. Secondly, the involvement of DNA2 in the repair of DNA damage induced by anticancer drugs makes it an attractive target in anti-cancer therapy. This is also supported by increased expression of DNA2 in cancer cell lines with various oncogenes activation. Moreover, this enhanced expression was positively correlated with atypical hyperplasia, a morphological feature of pre-malignant state. Furthermore, also tumor grade was correlated with the level of DNA2 expression. Therefore, DNA2 presents a potential as a cancer diagnostic, prognostic and prognostic marker as well as a target in cancer therapy. This seems especially important in estrogen-dependent cancers, as mutations in the *DNA2* gene were associated with breast and ovarian cancers. At least putative interaction of DNA2 with PARP1 makes it a protein important for SSBR and a candidate to target in synthetic lethality.

Relations of DNA2 with many aspects of cancer transformation and cancer clinics allow us to foresee further research on this protein with hope. As Judith Campbell, a leading scientist working on DNA2, said it can be “an Achilles heel for cancer cells” (http://www.bbe.caltech.edu/content/judith-l-campbell).

## Abbreviations

ATR	ATR serine/threonine kinase also known as ataxia telangiectasia and Rad3-related protein
Cdk1	Cyclin-dependent kinase 1
DNA2	DNA replication helicase/nuclease 2
DNA-PK_CS_	Catalytic subunit of DNA-dependent protein kinase
RPA	Replication protein A
FEN1	Flap endonuclease 1
WRN	Werner syndrome ATP-dependent helicase
BLM	Bloom syndrome protein
Pol γ, δ	DNA polymerase γ, δ
MRN	Mre11-Rad50-Nbs1 complex
NAM7	Nuclear accommodation of mitochondria 7
LP-BER	Long-Patch base excision repair
PIF1	Petite integration frequency 1
And-1	Acidic nucleoplasmic DNA-binding protein 1
RECQ1	ATP-dependent DNA helicase Q1
TOP1, 2	DNA topoisomerase I, II
ss, ds	Single-stranded, double-stranded
MGME1	Mitochondrial genome maintenance exonuclease 1
mtSSB	Mitochondrial single stranded DNA-binding protein
SUV3	Suppressor of var1 3-like
DSB	DNA double-strand break
G4	Guanine quartet
U2OS	Osteosarcoma cell line
HeLa	Ovarian cancer cell line
DSBR	DNA double-strand break repair
MMR	Mismatch repair
ICL	DNA interstrand cross-link
NHEJ	Non-homologous end joining
HRR	Homologous recombination repair
Sgs1	Slow growth suppressor 1
ExoI	Exonuclease I
SRCAP	Snf2-related CREB-binding protein activator protein
CtIP	C-terminal binding interacting protein
SMARCAD1	SWI/SNF-related matrix associated actin-dependent regulator of chromatin subfamily A containing DEAD/H box1
Swi/Snf	Switch/sucrose non-fermentable
BRCA1, 2	Breast cancer 1, 2
RAD51	Recombinase in homologous recombination
HELB	DNA helicase B
RMI1-2	RecQ mediated genome instability 1–2
Fun30	Function unknown now 30
γH2AX	Phosphorylated variant of the H2A histone
FA	Fanconi anemia
SP-, LP-BER	Short patch, long patch base excision repair
EXOG	5′ exo/endonuclease
SSBR	DNA single-strand break repair
H-RAS	Harvey rat sarcoma viral oncogene homolog
K-RAS	Kirsten rat sarcoma viral oncogene homolog
PARP1	Poly(ADP-ribose) polymerase 1
TP53	Tumor protein p53
